# The Role of MicroRNAs in Cancer Susceptibility

**DOI:** 10.1155/2013/591931

**Published:** 2013-03-19

**Authors:** Rodolfo Iuliano, Marco Flavio Michele Vismara, Vincenzo Dattilo, Francesco Trapasso, Francesco Baudi, Nicola Perrotti

**Affiliations:** ^1^Dipartimento di Medicina Sperimentale e Clinica, Università “Magna Graecia” di Catanzaro, 88100 Catanzaro, Italy; ^2^Unità Operativa di Genetica Medica, Università “Magna Graecia” di Catanzaro, 88100 Catanzaro, Italy; ^3^Dipartimento di Medicina Molecolare, Università di Roma “Sapienza”, 00161 Roma, Italy; ^4^Dipartimento di Scienze della Salute, Università “Magna Graecia” di Catanzaro, 88100 Catanzaro, Italy; ^5^Centro Oncologico Fondazione T. Campanella, 88100 Catanzaro, Italy

## Abstract

Single nucleotide polymorphisms (SNPs) are germline variations interspersed in the human genome. These subtle changes of DNA sequence can influence the susceptibility to various pathologies including cancer. The functional meaning of SNPs is not always clear, being, the majority of them, localized in noncoding regions. The discovery of microRNAs, tiny noncoding RNAs able to bind the 3′ untranslated region (UTR) of target genes and to consequently downregulate their expression, has provided a functional explanation of how some SNPs positioned in noncoding regions contribute to cancer susceptibility. In this paper we summarize the current knowledge of the effect on cancer susceptibility of SNPs included in regions related with miRNA-dependent pathways. Hereditary cancer comes up from mutations that occur in high-penetrant predisposing tumor genes. However, a considerable part of inherited cancers arises from multiple low-penetrant predisposing gene variants that influence the behavior of cancer insurgence. Despite the established significance of such polymorphic variants in cancer predisposition, sometimes their functional role remains unknown. The discovery of a new group of genes called microRNAs (miRNAs) opened an avenue for the functional interpretation of polymorphisms involved in cancer predisposition.

## 1. Biogenesis of MicroRNAs

MicroRNAs are an ample class (more than one thousand) of small (19–25 nucleotides) noncoding RNAs that downregulate the expression of target genes, binding mainly their 3′ untranslated region (3′UTR). Genomic regions containing microRNA genes are transcribed by RNA Polymerase II, and large RNA precursors, named pri-miRs (primary miRNAs), are generated [1]. The Pri-miRs are processed by a multiprotein complex in which the two core elements are Drosha, an enzyme with ribonuclease III activity, and Pasha (also known as DGCR8) an RNA-binding protein [2]. This processing results in the formation of a second precursor, called pre-miR, of ~70 nucleotides having a stem-loop structure with imperfect base pairings. The pre-miR is then exported by the complex Exportin5/RanGTP into the cytoplasm, where Dicer, a protein with ribonuclease III activity, cuts the RNA stem-loop structure leading to the formation of an miRNA duplex [1]. One strand of the duplex is selected, for thermodnamical reasons, to yield the mature miRNA while the other strand is degraded. Finally, the mature microRNA is loaded in a ribonuclease complex called RISC (RNA-induced silencing complex), containing the Argonaut (Ago 1–4) proteins and the GEMIN3 and GEMIN4 factors, where it pairs with the 3′UTR of target genes. The matching of miRNAs with their target genes causes the mRNA degradation or the inhibition of mRNA translation [3].

Single nucleotide polymorphisms (SNPs) related to miRNA genes, miRNA binding sites, or in genes of the miRNA processing machinery can affect the final level and function of miRNAs. This distinctive and relative new group of polymorphisms is called miRSNPs.

**Figure 1:**
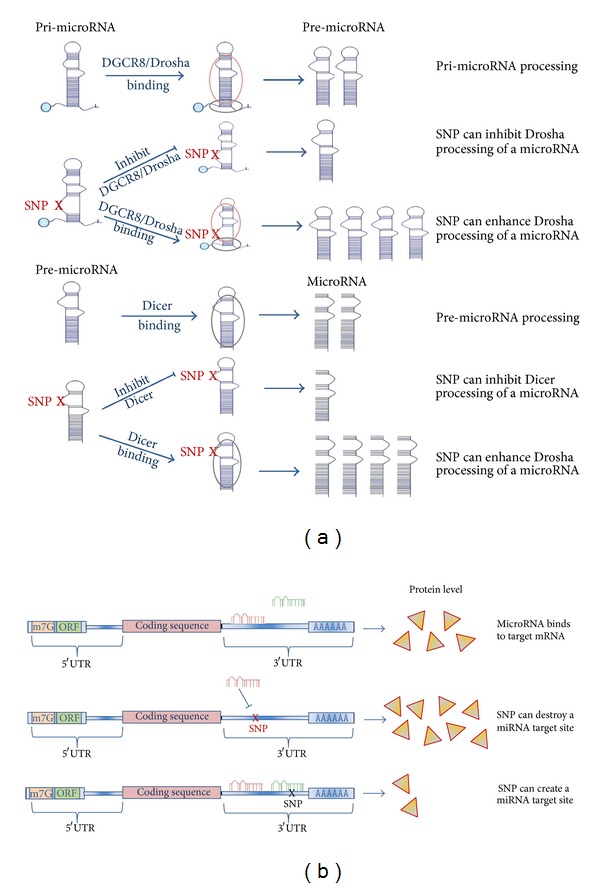
Schematic representation of influence of miRSNPs in microRNA processing (a) and in protein translation of microRNA target genes (b).

Target genes of microRNAs can be also oncogenes [4, 5] or tumor suppressor genes [6–8], and, hence, microRNAs are important players in carcinogenesis [9]. The presence of SNPs either in the genomic miRNA sequences or in the 3′UTR of cancer-related genes could influence miRNA-dependent regulation altering consequently tumor susceptibility (Figure 1) [10]. Here, we review miRSNPs studied for their relevance in the susceptibility to human cancer, indicating the associated risk expressed as odds ratio (OR) with 95% confidence interval (95% CI) (Table 1). 

## 2. SNPs in MicroRNA Genomic Regions

SNPs can span in the entire region of the pri-miR affecting the different steps of microRNA processing or the pairing with the 3′UTR of target genes. Therefore, the expression or the sequence of the mature miRNA could be impaired, and the regulation of all gene targeted by a given miRNA could be consequently affected.

Several SNPs in microRNA regions are currently intensively investigated in different cancer types but in some cases their significance remains to be established and further studies are required to clarify their possible role in cancer predisposition.

The SNP rs2910164 is located in the pri-miRNA sequence of miR-146a and has C and G allelic forms. It was first studied by Jazdzewski et al. whom found a significant different distribution of genotypes, in patients with papillary thyroid carcinomas compared with normal subjects, being the GC genotype associated with an increased risk of papillary thyroid carcinoma (OR = 1.62; 95% CI: 1.3–2.0; *P* = 0.000007) [11]. Furthermore, the authors demonstrated that in heterozygotes the expression of mature miR-146a is reduced. Wang et al. showed that the C allele of SNP rs2910164 is associated with significantly decreased risk of bladder cancer (OR = 0.80; 95% CI: 0.71–0.90), and GC/CC genotypes confer a significantly reduced risk of recurrence, compared with the GG genotype. The same authors demonstrated by functional studies that the miR-146a rs2910164 C allele inhibits proliferation in bladder cancer cells [12]. In a case-control study, Lung et al. showed that CC genotype of rs2910164 was associated with an increased risk of nasopharyngeal carcinoma (NPC) (GC + GG versus CC, OR = 0.49; 95% CI: 0.35–0.69). The authors also demonstrated that passenger strand miR-146a*C in NPC is significantly increased in CC genotype, resulting in the regulation of a set of target genes [13]. The allelic variants of rs2910164 were evaluated in familial breast and ovarian cancers in BRCA1/BRCA2-negative patients in a study which suggested that the polymorphism may impact on the age of cancer onset. In fact, subjects with GC or CC genotypes developed tumors at younger age compared with individuals carrying the GG genotype [14]. However, a large study of breast cancer cases negative for disease-causing mutations or unclassified variants in BRCA1 and BRCA2 showed no associations between rs2910164 genotype and breast cancer susceptibility [15]. Another study showed also lack of association of the rs2910164 SNP with breast cancer risk in a series of BRCA1 and BRCA2 mutation carriers [16]. In a meth-analysis study, a stratified analysis by ethnicity showed that the rs2910164 polymorphism is associated with increased breast cancer risk among Europeans in a recessive model (CC versus GC + GG: OR = 1.31; 95% CI: 1.05–1.65) [17]. In another meta-analysis was shown that SNP rs2910164 is not associated with the risk of hepatocellular carcinoma [18]. Thus, the C allele of rs2910164 seems to be associated with cancer risk in a cancer type specific manner, but further studies are required to better clarify this matter. In addition, other functional studies should define the role of the polymorphism in the expression of miR-146a. 

The SNP rs11614913 is located in the pre-miRNA region of miR-196a2 and has two allelic forms, T and C. Hu et al. found that in nonsmall cell lung cancer patients who were homozygous CC at SNP rs11614913, risk of death significantly increased (hazard ratio HR = 1.76; 95% CI: 1.34–2.32). The genotype rs11614913 CC was associated with a significant increase in mature hsa-mir-196a expression without changes in levels of the precursor, suggesting an enhanced processing of the pre-miRNA [19, 20]. The miR-196a2 CC genotype was also associated with reduced survival in patients with pharyngeal tumors [21] increased colorectal cancer risk [22] and increased breast cancer risk [23] compared with the TT/CT genotype. However, in breast cancer, Catucci et al. found no significant increased risk in rs11614913 CC homozygous patients [15]. A meta-analysis of 15 studies showed that individuals with the TC/CC genotypes are associated with higher cancer risk than those with the TT genotype (OR = 1.18; 95% CI: 1.03–1.34; *P* < 0.001) supporting the hypothesis that hsa-miR-196a2 rs11614913 polymorphism may contribute to cancer susceptibility [24].

The SNP rs6505162 is an A/C polymorphism located in the pre-miRNA region of miR-423. Ye et al. systematically evaluated the effects of 41 genetic variants in 26 miRNA-related genes on esophageal cancer risk. The most relevant finding was the association of rs6505162 SNP with reduced esophageal cancer risk, following an additive model (OR = 0.64; 95% CI: 0.51–0.80, *P* < 0.0001), being the C allele less represented in cases than in controls [25]. For the same polymorphism, a study that was performed on Caucasian Australian women showed that CC genotype confers a reduced risk of breast cancer development (OR = 0.50; 95% CI: 0.27–0.92) [26]. In a sample of Chinese patients with surgically resected colorectal adenocarcinoma, homozygous CC genotypes of SNP rs6505162 were significantly associated with both the overall survival (HR = 2.12; 95% CI: 1.34–3.34, *P* = 0.001) and the recurrence-free survival (HR = 1.59; 95% CI: 1.08–2.36) [27].

The SNP rs3746444, having the two alleles A and G, is located in the pre-miR region of miR-499. In a case-control study on a Chinese population, the variant G was associated with a significant increased risks of breast cancer (OR = 1.25; 95% CI: 1.02–1.51) in a dominant genetic model [23]. This association was not confirmed by the study of Catucci et al. performed on a Caucasian population [15]. In a meta-analysis study the rs3746444 polymorphism was significantly associated with breast cancer risk in Asian population, being the G allele responsible for the increased risk (OR = 1.10; 95% CI: 1.01–1.20) [28]. Moreover, the rs3746444 polymorphism was associated with a significant increased risk (OR = 1.98; 95% CI: 1.36–2.98; *P* = 0.0004) of cervical squamous cell carcinoma, following an overdominant model [29]. 

SNP rs4919510 is located in pre-miRNA region of miR-608 and has C and G alleles. The relevance of this polymorphism has been studied in patients with colorectal cancer (CRC). In CRC patients receiving first-line fluoropyrimidine-based chemotherapy several SNPs located in miRNA regions were genotyped, the rs4919510 resulted associated with increased risk for both recurrence and death (HR = 2.72; 95% CI: 1.38–5.33 and HR = 3.53; 95% CI: 1.42–8.73) [30]. These findings were confirmed by an independent study, in which rs4919510 was associated with altered recurrence-free survival only in patients receiving chemotherapy but not in those without chemotherapy [27]. In another study, there was no significant association found between rs4919510 and colorectal cancer risk, but the GG genotype was associated with an increased risk of death in Caucasians and with a reduced risk of death in African Americans [31]. Recently, rs4919510 was studied in breast cancer patients, and the G-allele was specifically associated with an increased risk of HER2-positive subtype (OR = 1.62; 95% CI: 1.23–2.15) [32].

The SNP rs895819 is located in the pre-miRNA-27a and has A and G alleles. The role of this SNP was first investigated in a German familial breast cancer study cohort, in which SNPs related to other microRNAs were also analyzed. The G allele of SNP rs895819 resulted significantly less frequent in cases than in controls, indicating a lower familial breast cancer risk for patients carrying this variant (OR = 0.88; 95% CI: 0.78–0.99) [33]. The same conclusion was not confirmed in an Italian breast cancer cohort with similar characteristics [34]. The discordance between these two studies was further investigated, and it is probably due to technical aspects concerning the genotyping method used [35]. In a case-control study on renal carcinoma, individuals carrying AG/GG genotypes of SNP rs895819 had a statistically significant lower susceptibility in the development of renal cancer (OR = 0.71; 95% CI: 0.56–0.90) than individuals with AA genotype [36]. In another case-control study which was performed to investigate the role of SNP rs895819 in gastric cancer susceptibility, subjects with the variant genotypes (AG + GG) showed an increased risk of gastric cancer relative to AA carriers (OR = 1.48; 95% CI: 1.06–2.05) [37]. Moreover, patients with AA genotypes showed higher levels of miR-27a compared with AG and GG genotypes, and the miR-27a levels resulted inversely correlated with the expression of ZBTB10, an miR-27a target gene.

The SNP rs213210 is a C/T variation in the region of miR-219-1. This polymorphisms was associated with increased risk in esophageal cancer with a dominant model (OR = 1.75; 95% CI: 1.10–2.80) [25] and increased risk of death in patients with colorectal cancer receiving first-line fluoropyrimidine-based chemotherapy (HR = 3.22; 95% CI: 1.70–6.10) [30].

## 3. SNPs in microRNA-Targeted 3′UTRs

SNP variants located in 3′UTRs can destroy or create miRNA binding sites influencing tumor susceptibility. In order to evaluate the importance of the SNPs in cancer susceptibility, both microRNAs and target genes should have relevance in the tumor type considered. 

To establish the role in cancer susceptibility of SNPs located in the 3′UTR of miRNA-targeted genes, beyond association studies, two kinds of functional assays should be performed. First, both SNP allelic forms of the 3′UTR should be cloned downstream of a reporter gene (usually a luciferase) and in cells cotransfected with the interacting micro-RNA, and the alternative 3′UTRs forms the quantified luciferase activity should be significantly different. Second, in biological samples the levels of protein or messenger RNA of the different allelic forms should be dependent on the expression of the interacting microRNA. 

Among the SNPs located in the 3′UTRs, the most studied is the rs61764370, which is located in the *KRAS* gene. It has G and T alleles and influences the binding of *KRAS* with let-7. Remarkably, both *KRAS* and let-7 are important in cancer development being *KRAS* a well-known oncogene and let-7 a microRNA that acts as tumor suppressor gene [4]. Therefore, the rs61764370 polymorphism fulfills the criteria of relevance in cancer predisposition. This polymorphism was studied for the first time by Chin et al. in nonsmall lung cancer patients [38]. These authors showed that the less common G variant of rs61764370 disrupts significantly the binding of microRNA let-7 to the 3′UTR of *KRAS*, increasing *KRAS* expression. In addition, in that study was found that subjects (smoking < 40 pack/year) with the GG + GT genotypes have an increased risk (OR = 1.36; 95% CI: 1.07–1.73; *P* = 0.01) of nonsmall lung cancer. After this first study, G variant of rs61764370 was found associated with higher risk in breast cancer [39], colorectal cancer [40], melanoma [41], oral cancers [42], and ovarian cancer [43]. Conversely, other authors did not find significant association of rs61764370 with cancer risk in colorectal cancer [44], nonsmall lung cancer [45], and ovarian cancer [46]. Thus, the relevance of rs61764370 in cancer predisposition is still debated and deserves further investigations. 

The SNP rs334348 is located in the 3′UTR of *TGFBR1* gene and has A and G variants. Nicoloso et al. found that subjects with AG genotype has higher susceptibility to familiar breast cancer (OR = 2.2; 95% CI: 1.29–4.07; *P* = 0.005) [47]. Functionally, it was shown that the G allele of rs334348 is targeted with higher efficiency by miR-628-5p than the A allele. This was demonstrated by luciferase assay using the cloned alternative forms of *TGFBR1* 3′UTR and by western blot using breast cancer cell lines with different genotypes. Notably, the expression of TGFBR1 influences significantly the colorectal cancer risk [48].

The SNP rs3134615 is in the 3′-UTR of *MYCL1*, within a miR-1827 binding site, and has G and T alleles. In a case-control study, Xiong et al. showed that subjects with TG and TT genotypes have an increased risk (OR = 2.08; 95% CI: 1.39–3.21; *P* = 0.0004) for the development of small-cell lung cancer [49]. Moreover, the same authors demonstrated that the T allele significantly impairs the interaction of miR-1827 with the 3′UTR of *MYCL1*, reducing miR-1827 dependent inhibition of *MYCL1* expression.

The SNP rs7963551 is located in the 3′UTR of *RAD52* and has C and A variants. *RAD52* encodes a protein involved in homologous recombination repair. In a study performed to evaluate the relevance of rs7963551 on breast cancer susceptibility, the C allele was associated with reduced cancer risk (OR = 0.84; 95% CI: 0.75–0.95). Moreover, a reduced inhibition of *RAD52* expression of C allele, probably due to a weakened binding capacity of hsa-let-7 to 3′-UTR of *RAD52*, was demonstrated by luciferase activity assay in MCF-7 cell line [50]. 

The rs16917496 is a C/T variation located in the 3′UTR of *SET8* within the miR-502 binding site, having the C allele a perfect match in the seed region of miR-502. The expression of *SET8* in breast tumor tissues of patients with a CC genotype is significantly lower than in patients with TT genotype, and, importantly, the age of breast cancer onset depends on the number of C alleles, being significantly lower in C allele carriers (*P* = 0.022) [51]. This is in agreement with the role played by *SET8*, a methyltransferase that acts on p53 increasing its proapoptotic potential [52]. It was found that the *SET8* CC genotype confers also longer survival to patients with hepatocellular carcinoma [53] and with small-cell lung cancer [54], and it is associated with a decreased risk of epithelial ovarian cancer [55].

The rs1049253 is a C/T variation located in the 3′UTR of *CASP3* gene within the binding site of miR-885-5p. *CASP3* belongs to the caspase family genes encoding key effector enzymes involved in cell apoptosis. Downregulation of caspases affects programmed cell death allowing tumor cell proliferation. A study on 7 SNPs located in the 3′UTR of caspase genes was performed to assess their possible association with squamous cell carcinoma of the head and neck. The genotypes CC/CT rs1049253 resulted associated with significantly increased cancer risk (OR = 1.29; 95% CI: 1.07–1.56), but no associations were found for the other 6 SNPs. Moreover, the rs1049253 CC genotype was associated with reduced levels of *CASP3* mRNA compared with the TT genotype, and C allele resulted in stronger down-regulation than T allele of the *CASP3* expression determined with miR-885-5p mimic transfection and luciferase assay [56].

In a study of Landi et al. [57], SNPs located in the miRNA binding sites of 3′UTR of genes relevant in the pathogenesis of colorectal cancer (CRC) were computationally tested for their ability to affect the binding of the miRNA with its target. Eight polymorphisms were further studied by case-control studies and two of them, rs17281995 and rs1051690, located in the *CD86* and *INSR* genes respectively, resulted significantly associated with CRC risk. In a subsequent paper [58], the same authors extended the study of rs17281995 and rs1051690 polymorphisms in CRC risk, analyzing an additional population (OR = 2.93; 95% CI: 1.29–6.67, for rs17281995 and OR = 2.06; 95% CI: 1.19–3.56, for rs1051690, in the pooled samples, following a codominant model).

The SNP rs1044129 is a G/A variation located in the 3′UTR of *RYR3* gene in the miR-367 binding site. *RYR3* encodes a protein that forms a calcium channel which is relevant for cell growth and migration of breast cancer cells. In a case-control study, AA genotype carriers showed a significant higher breast cancer risk (OR = 1.26, 95% CI: 1.03–1.54) than G allele carriers (GA + GG genotypes) [59]. Moreover, breast cancer patients with AA genotype had a significantly higher progression-free survival (*P* = 0.036) than patients carrying the G allele. The authors also demonstrated by functional assays that the A allele is more repressed by miR-367 than the G allele. 

*HOXB5*, a member of the *HOX* gene family, has a SNP (1010A/G) in its 3′UTR in the binding site of miR-7. In a comprehensive study, Luo et al. [60] first showed the importance of *HOXB5* in the pathogenesis of bladder cancer demonstrating that inhibition of its expression decreases bladder cell proliferation and tumorigenicity. Then, the authors found that subjects carrying the G allele (AG + GG genotypes) have a higher bladder cancer risk (OR = 1.48; 95% CI: 1.07–2.06) compared with AA genotype carriers. In agreement with the above results, the authors showed by luciferase assay that the 1010A allele is repressed with higher efficiency by miR-7 than the 1010G allele.

The rs4245739 is an A/C variation located in the 3′UTR of *MDM4* in an miR-191 binding site. Overexpression of MDM4 protein promotes tumorigenesis by the negative regulation of P53 activity. In experiments performed in ovarian cancer cells, Wynendaele et al. [61] showed a stronger inhibitory effect of miR-191 on the A allele compared with C allele. Furthermore, the authors showed that patients with ER negative ovarian cancer and homozygous for the A allele have an increased risk of tumor-related death (HR = 5.5; 95% CI: 1.5–20.5).

## 4. SNPs in Genes Encoding microRNA Processing Proteins

Polymorphisms which influence the expression of proteins involved in miRNA biogenesis pathway may affect miRNA-mediated regulation within the cell and may contribute to the genetic variation observed in specific phenotypes [62]. Several studies focused on miRSNPs of the silencing machinery, and here we summarize this knowledge. 

A Korean study [63] of 2010 evaluated *AGO1*, *AGO2*, *TNRC6A*, *TNRC6C*, *TARBP2* and *XPO5* mutations in colorectal (CRC) and gastric cancers (GC), with or without microsatellite instability. Data of this study indicate that frameshift mutations in *AGO2* and *TNRC6A* and their losses of expression are common in GCs and CRCs with microsatellite instability and suggest that these alterations may contribute to the cancer development by deregulating miRNAs.

In a study on renal cancer [64], 40 SNPs from 11 miRNA processing genes (*DROSHA*, *DGCR8*, *XPO5*, *RAN*, *DICER1*, *TARBP2*, *AGO1*, *AGO2*, *GEMIN3*, *GEMIN4*, and *HIWI*) were genotypized. Two SNPs in the *GEMIN4* gene were significantly associated with altered renal cell carcinoma risk. The variant-containing genotypes of Asn929Asp and Cys1033Arg significantly reduced risk, with odds ratios of 0.67 (95% CI: 0.47–0.96) and 0.68 (95% CI = 0.47–0.98), respectively. Haplotype analysis showed that a common haplotype of *GEMIN4* was associated with a significant reduction in the risk of renal cell carcinoma (OR = 0.66; 95% CI: 0.45–0.97). 

The same research group, in a successive study [65], took a polygenic approach to evaluate the effects of 41 potentially functional SNPs in miRNAs-related genes on survival and recurrence among renal cell carcinoma (RCC) patients. In single-SNP analysis, seven SNPs were identified significantly associated with RCC survival and five SNPs with recurrence. The most significant associations were SNPs in *GEMIN4* with the variant alleles of both rs7813 and rs910925 associated with 1.74-fold (95% CI: 1.15–2.62) increased risk of death, whereas the variant allele of rs3744741 conferred a decreased risk of death (HR = 0.39; 95% CI: 0.19–0.77). Haplotypes of *DICER* and *DROSHA* were also associated with altered patient survival and recurrence.

To test the hypothesis that adverse alleles in miRNA-related genes may influence the risk for esophageal cancer, the associations between esophageal cancer risk and 41 potentially functional SNPs in 26 miRNA-related genes were assessed, in a case-control study [25]. A common haplotype of the *GEMIN4* gene was associated with a significantly reduced risk of esophageal cancer (OR = 0.65; 95% CI: 0.42–0.99).

In a recent study [66], the authors used genotype data available from a previous case-control study to investigate association between common genetic variations in miRNA biogenesis pathway genes and breast cancer survival. They investigated the possible associations between 41 SNPs and both disease-free survival (DFS) and overall survival (OS) among 488 breast cancer patients. Two SNPs in AGO2 (rs11786030 and rs2292779) and *DICER1* rs1057035 were associated with both DFS and OS. Two SNPs in *HIWI* (rs4759659 and rs11060845), and *DGCR8* rs9606250 were associated with DFS, while *DROSHA* rs874332 and *GEMIN4* rs4968104 were associated with OS only. The most significant associations were observed in variant allele of *AGO2* rs11786030 with 2.62-fold increased risk of disease progression (95% CI: 1.41–4.88) and in minor allele homozygote of *AGO2* rs2292779 with 2.94-fold increased risk of death (95% CI: 1.52–5.69).

A recent study was performed in order to evaluate the role of miR-SNPs of *GEMIN4* in prostate cancer [67]. The high-resolution melting method was used to genotype seven polymorphisms (rs7813, rs4968104, rs3744741, rs2740348, rs1062923, rs910925, and rs910924) in the *GEMIN4* gene. Patients carrying the variant heterozygote GC genotype in the rs2740348 were at a 36% decreased risk of prostate cancer (OR = 0.64; 95% CI: 0.42–0.99). In addition, subjects carrying the homozygote TT genotype in the rs7813 had a significantly increased risk of prostate cancer (OR = 2.53; 95% CI = 1.07–6.28). Two common haplotypes were found to be associated with decreased risk of prostate cancer. In the subgroup analysis, higher risk of more severity of prostate cancer (clinical stage III and IV) was observed in individuals with the rs7813 TT genotype (OR = 2.64; 95% CI: 1.02–7.64), while lower risk of more severity of prostate cancer was observed in individuals with the rs3744741 T allele (OR = 0.69; 95% CI: 0.50–0.96).

In a study on a sample of nonsmall-cell lung cancer patients [68], three miR-SNPs in microRNA-processing machinery components were examined, and the time to recurrence (TTR) according to miR-SNP genotypes was evaluated. Significant differences in TTR were found for *XPO5* rs11077 (median TTR: 24.7 months for the AA genotype versus 73.1 months for the AC or CC genotypes; *P *= 0.029). In multivariate analyses, the *XPO5* rs11077 AA genotype (OR = 1.77; 95% CI: 1.07–2.91) emerged as independent variable influencing TTR. 

In a recent case-control study on head and neck cancer (HNC) [69], three SNPs at miRNA binding sites of miRNA processing genes were genotyped (rs1057035 in 3′UTR of *DICER*, rs3803012 in 3′UTR of *RAN,* and rs10773771 in 3′UTR of *HIWI*). Although none of the SNPs was significantly associated with overall risk of HNC, rs1057035 in 3′UTR of *DICER* was associated with a significantly decreased risk of oral cancer (TC/CC versus TT, OR = 0.65; 95% CI: 0.46–0.92). Furthermore, luciferase activity assay showed that rs1057035 variant C allele led to significantly lower expression levels of DICER as compared to the T allele, which may be due to the higher inhibition of hsa-miR-574-3p on *DICER* mRNA. These findings indicated that rs1057035 located at 3′UTR of *DICER* may contribute to the risk of oral cancer by affecting the binding of miRNAs to *DICER*. Large-scale and well-designed studies are warranted to validate these findings.

A sample of patients with chemosensitive multiple myeloma intensified with autologous stem cell transplantation was studied in a longitudinal study [70]. The SNP of miRNA biogenesis pathway evaluated was the rs11077 of *XPO5*. Overall survival was significantly longer in patients with C/C or A/C variant in *XPO5* rs11077 (*P* = 0.012). A significant longer progression-free survival for this SNP (*P* = 0.013) was also found. 

## 5. Conclusions

The study of polymorphisms, affecting miRNA-dependent pathways and involved in cancer susceptibility, is rapidly growing, and in the near future probably other SNPs will be investigated, and the SNPs mentioned in this paper will be further evaluated. The acquisition of new data in different populations has a paramount importance to establish the real contribution of each polymorphism to the cancer risk in this promising area.

## Figures and Tables

**Table 1:** List of miRSNPs evaluated in cancer susceptibility studies.

SNP category	Cancer type	SNP	Gene	OR (95% CI)	Reference
miRNA sequence					

	Any cancer	rs11614913	miR-196a2	1.18 (1.03–1.34)	[24]

	Bladder cancer	rs2910164	miR-146-a	0.80 (0.71–0.90)	[12]

		rs895819	miR-27a	0.88 (0.78–0.99)	[33]
		rs2910164	miR-146-a	1.31 (1.05–1.65)	[17]
		rs11614913	miR-196a2	1.23 (1.02–1.48)	[23]
	Breast cancer	rs6505162	miR-423	0.50 (0.27–0.92)	[26]
		rs3746444	miR-499	1.25 (1.02–1.51)	[23]
		rs3746444	miR-499	1.10 (1.01–1.20)	[28]
		rs4919510	miR-608	1.62 (1.23–2.15)	[32]

	Cervical cancer	rs3746444	miR-499	1.98 (1.36–2.89)	[29]

		rs213210	miR-219-1	3.22 (1.70–6.10)	[30]
	Colorectal carcinoma	rs6505162	miR-423	2.12 (1.34–3.34)	[27]
		rs4919510	miR-608	2.72 (1.38–5.33)	[30]

	Esophageal cancer	rs213210	miR-219-1	1.75 (1.10–2.80)	[25]
		rs6505162	miR-423	0.64 (0.51–0.80)	[25]

	Gastric cancer	rs895819	miR-27a	1.48 (1.06–2.05)	[37]

	Nasopharyngeal carcinoma	rs2910164	miR-146-a	0.49 (0.35–0.69)	[13]

	Non-small cell lung cancer	rs11614913	miR-196a2	1.76 (1.34–2.32)	[19]

	Papillary thyroid carcinoma	rs2910164	miR-146-a	1.62 (1.3–2.0)	[11]

	Renal carcinoma	rs895819	miR-27a	0.71 (0.56–0.90)	[36]

Binding site					

	Bladder cancer	1010A/G	*HOXB5 *	1.48 (1.07–2.06)	[60]

		rs61764370	*KRAS *	1.47 (1.05–2.06)	[39]
	Breast cancer	rs7963551	*RAD52 *	0.84 (0.75–0.95)	[50]
		rs1044129	*RYR3 *	1.26 (1.03–1.54)	[59]
		rs334248	*TGFBR1 *	2.2 (1.29–4.07)	[47]

	Colorectal carcinoma	rs17281995	*CD86 *	2.93 (1.29–6.67)	[58]
		rs1051690	*INSR *	2.06 (1.19–3.56)	[58]

	Non-small cell lung cancer	rs61764370	*KRAS *	1.36 (1.07–1.73)	[38]

	Ovarian cancer	rs61764370	*KRAS *	1.67 (1.09–2.57)	[43]
		rs4245739	*MDM4 *	5.5 (1.5–20.5)	[61]

	Small-cell lung cancer	rs3134615	*MYCL1 *	2.08 (1.39–3.21)	[49]
		rs16917496	*SET8 *	0.45 (0.22–0.94)	[54]

	Squamous cell carcinoma of the head and neck	rs1049253	*CASP3 *	1.29 (1.07–1.56)	[56]

Biogenesis pathway					

	Breast cancer	rs11786030	*AGO2 *	2.62 (1.41–4.88)	[66]
		rs2292779	*AGO2 *	2.94 (1.52–5.69)	[66]

	Head and neck cancer	rs1057035	*DICER *	0.65 (0.46–0.92)	[69]

	Non-small cell lung cancer	rs11077	*XPO5 *	1.77 (1.07–2.91)	[68]

		rs2740348	*GEMIN4 *	0.64 (0.42–0.99)	[67]
	Prostate cancer	rs7813	*GEMIN4 *	2.53 (1.07–6.28)	[67]
		rs3744741	*GEMIN4 *	0.69 (0.50–0.96)	[67]

		rs2740348	*GEMIN4 *	0.67 (0.47–0.96)	[64]
	Renal carcinoma	rs7813	*GEMIN4 *	0.68 (0.47–0.98)	[64]
		rs910925	*GEMIN4 *	1.74 (1.15–1.62)	[65]
		rs3744741	*GEMIN4 *	0.39 (0.19–0.77)	[65]
